# Arterial and Venous Thromboembolism Associated With Whippet-Induced Vitamin B12 Deficiency

**DOI:** 10.7759/cureus.84643

**Published:** 2025-05-22

**Authors:** Noor Ul Ain Shahid, Noman Saleem, Anisha Dave, Mahmoud Othman, Cortney V Jones

**Affiliations:** 1 Internal Medicine, Ameer-ud-Din Medical College/Lahore General Hospital, Lahore, PAK; 2 Internal Medicine, Detroit Medical Center/Sinai Grace Hospital, Detroit, USA; 3 Interventional Cardiology, Detroit Medical Center/Wayne State University, Detroit, USA; 4 Hematology and Oncology, Detroit Medical Center/Wayne State University, Detroit, USA

**Keywords:** arterial venous thromboembolism, methylmalonic acid, saddle pulmonary embolism, serum homocysteine, subacute combined degeneration of the spinal cord, whippets

## Abstract

Recreational whippet (nitrous oxide) use is increasing among young adults due to its euphoric effects. However, this practice is associated with vitamin B12 deficiency, which can manifest in a wide range of symptoms. This case report details a case of a 31-year-old female presenting with both arterial and venous thromboembolism secondary to vitamin B12 deficiency induced by whippet use. Initially presenting as a stroke code, initial workup was unremarkable. Later, the patient exhibited right-sided weakness within the first 12 hours and was found to have a perfusion mismatch defect in the left frontal-parietal lobe. Further investigation revealed simultaneously left carotid artery stenosis and occlusion, along with a pulmonary embolism. Treatment included embolectomy and stent placement. Laboratory results confirmed low vitamin B12 levels, elevated methylmalonic acid, and elevated homocysteine. Common causes that were linked with both arterial and venous thromboembolism were ruled out, including lupus, protein C and S deficiency, antithrombin 3 and factor V laden mutation. Following vitamin B12 supplementation and rehabilitation, the patient showed improvement in her aphasia and right-sided weakness.

## Introduction

Recreational whippet (nitrous oxide) use among young adults is increasing, driven by the pursuit of short-lived euphoria (five to 20 seconds) [1]. While nitrous oxide has legitimate applications in the food industry, its recreational misuse can lead to vitamin B12 deficiency. Existing literature primarily links nitrous oxide-induced B12 deficiency to glial cell dysfunction, resulting in central nervous system demyelination and subacute combined degeneration of the spinal cord [2-4]. Both methylmalonic acid and homocysteinemia have been linked as independent risk factors of cardiovascular disease and arterial and venous thromboembolism [5]. Homocysteineimia primarily increases risk of thromboembolism by interfering with the activation of factor Va, increasing the level of protein C, expression of tissue factor, and suppression of sulfate heparin [6]. This case presents a patient with both arterial and venous thromboembolism associated with vitamin B12 deficiency secondary to recreational nitrous oxide use.

## Case presentation

**Figure 1:**
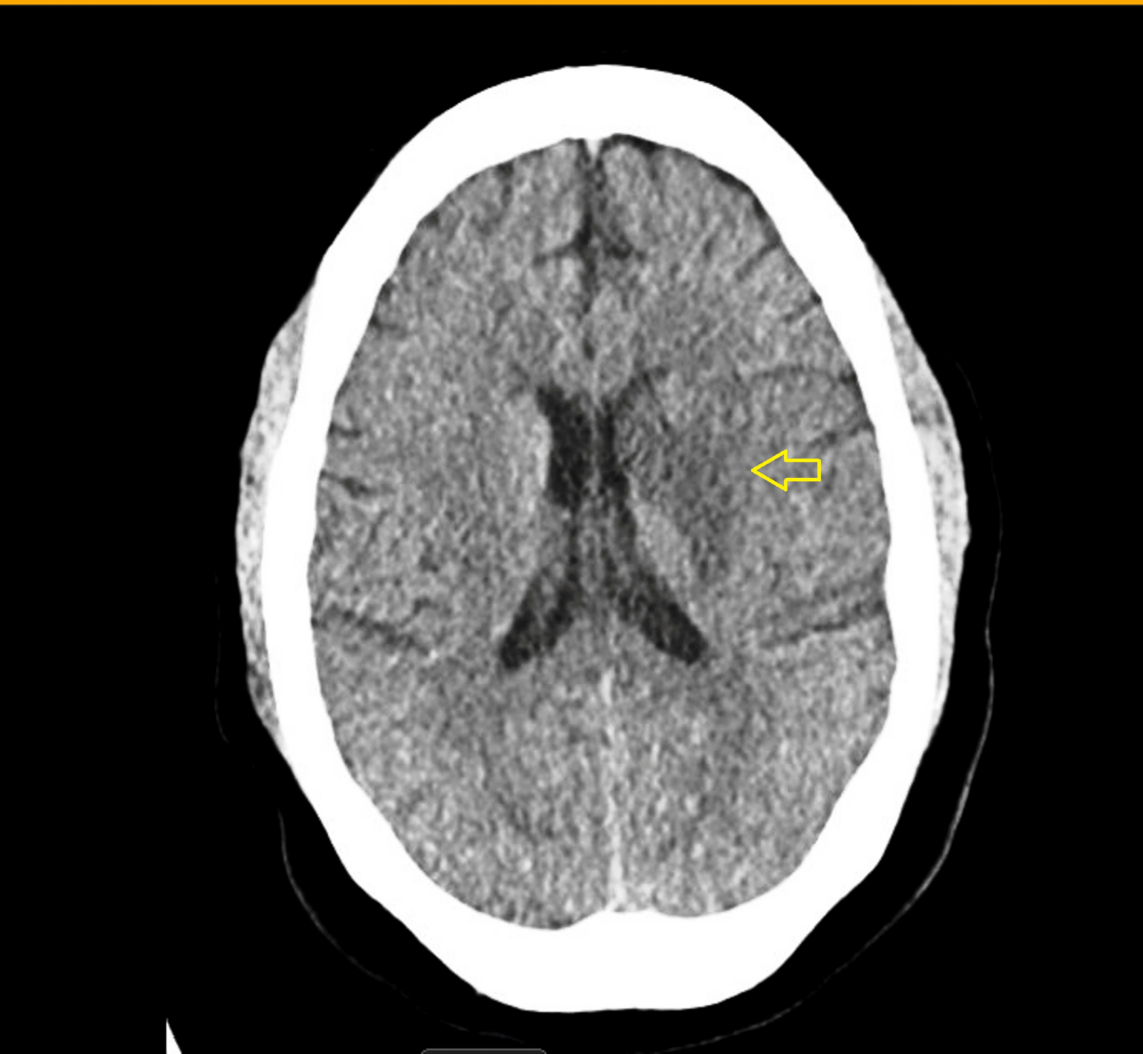
CT Head without contrast: Left frontal-parietal lobe infarct within the middle cerebral artery (MCA) territory (yellow arrow). This will lead to the right side hemiplegia, hemisensory loss, gaze deviation and neglect syndrome.

A 31-year-old African American female with a documented history of polysubstance use disorder (cocaine, whippets, cannabis), chronic inflammatory demyelinating polyneuropathy/Guillain-Barré syndrome, bilateral foot drop, and a prior pulmonary embolism (not currently on anticoagulation therapy due to noncompliance) was transported to the emergency department (ED) by emergency medical services (EMS) secondary to abnormal behavior and unresponsiveness. EMS personnel reported discovering the patient in an altered state, exhibiting some right-sided lower facial droop. Initial EMS evaluation did not indicate a drug overdose. During attempts to establish intravenous (IV) access in the ED, the patient became combative and demonstrated movement in all four extremities. Due to her agitated state, she was administered 5 mg of Versed, and initial diagnostic procedures were conducted. Laboratory analysis revealed elevated lactate levels and platelet clumping on complete blood count (CBC), and it was confirmed on peripheral blood smear. Repeat lab sample showed normal platelet count. The likely reason for elevation in serum lactate is disruption in the metabolic process related to nitrous oxide use. Computed tomography (CT) imaging of the head was unremarkable. Considering her history of polysubstance use disorder, she was admitted to the general medical floor with a diagnosis of toxic encephalopathy. Following subsequent examination, our medical team observed adequate strength in all extremities and absence of the right facial droop that was reported by the EMS, although the patient demonstrated resistance to the examination and responded to questions intermittently. She was also administered 2 mg of Ativan by the ED physician due to concerns regarding potential catatonia in the resus bay. During the night on day one, she subsequently developed right-sided hemiparesis, which necessitated the activation of a stroke code. A CT scan of the head without contrast enhancement revealed a substantial perfusion mismatch defect in the left frontal-parietal lobe within the middle cerebral artery (MCA) territory, suggestive of ischemic penumbra, accompanied by a smaller core perfusion defect (Figure 1).

**Figure 2:**
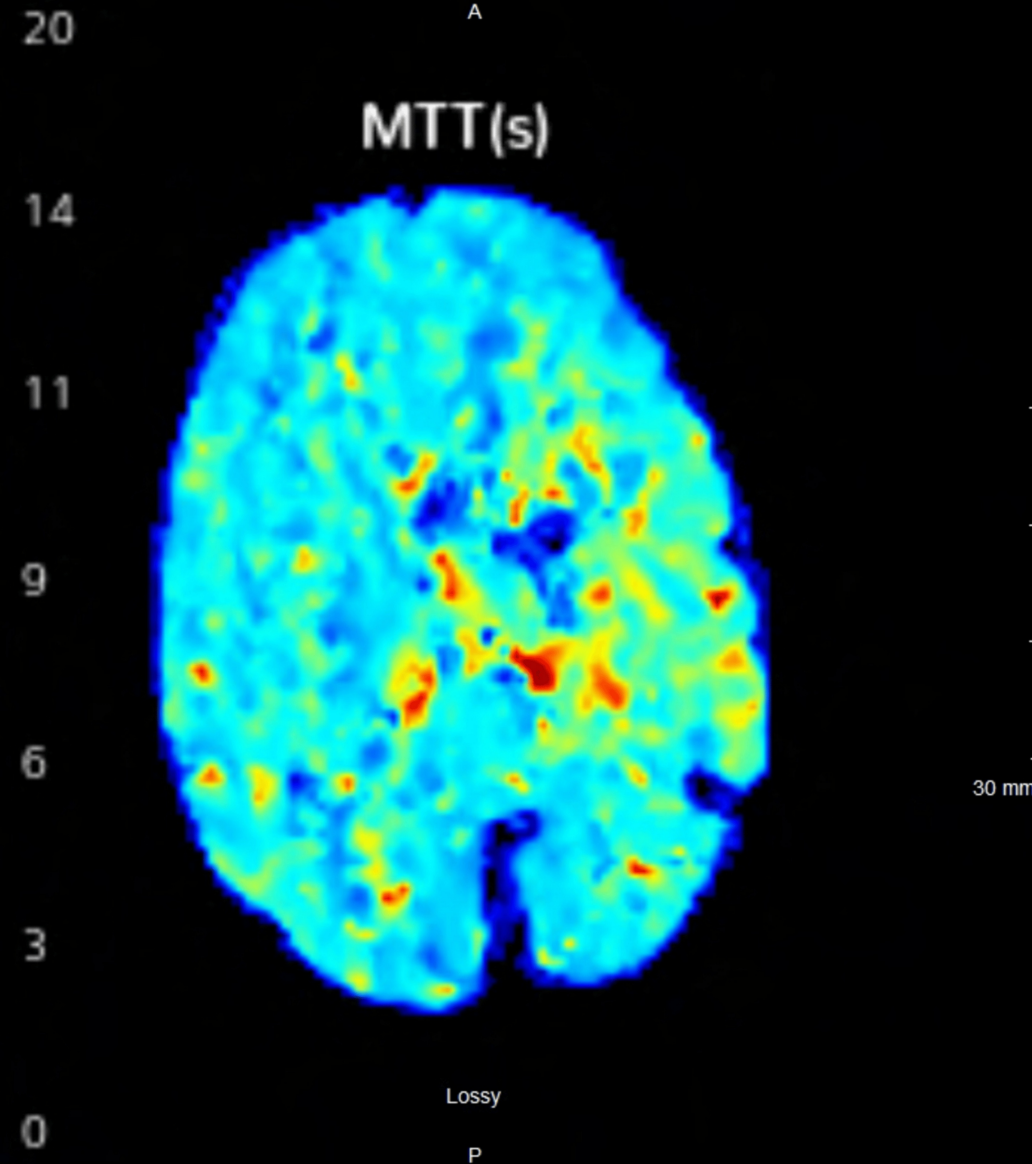
CT perfusion showing mismatch perfusion defect in left frontal parietal region. MTT: mean transit time

CT angiography (CTA) of the head and neck demonstrated a soft atherosclerotic plaque at the left common carotid bifurcation with 60% stenosis, diffuse narrowing of the cervical and proximal petrous segment of the left internal carotid artery with distal occlusion, and occlusion of the left MCA with minimal reconstitution from the left anterior cerebral artery. CT perfusion studies corroborated the mismatch perfusion defect, indicating a mismatch volume of 51 cc and a mismatch pressure of 6.7 (Figure 2).

**Figure 3:**
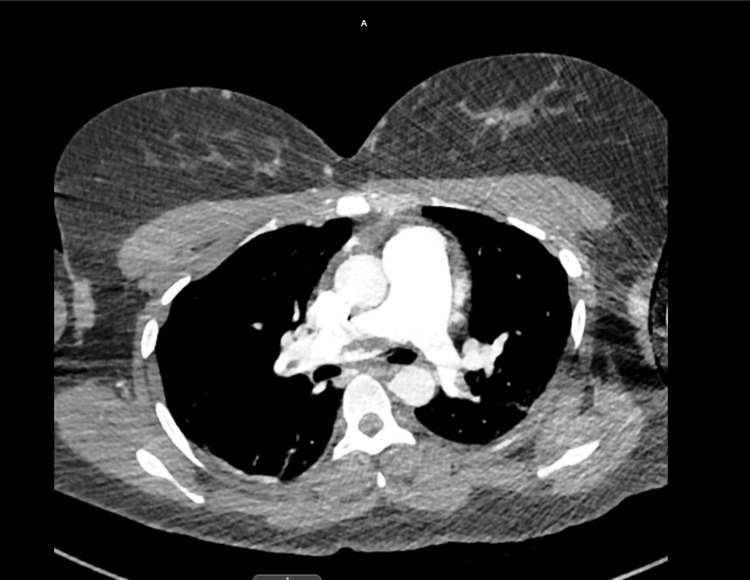
CT thorax with pulmonary embolism protocol showed saddle pulmonary embolism

Consultations with Neuroendovascular and Neurosurgery specialists were obtained. The patient subsequently underwent embolectomy of the left common carotid artery, left internal carotid artery, and left MCA, in conjunction with left carotid artery stent placement. CT imaging of the neck incidentally revealed a pulmonary embolism, which was subsequently confirmed by CT thorax imaging, demonstrating a saddle embolism without evidence of right heart strain (Figure 3). 

Following consultation with the cardiology service, IV heparin was initiated, and the patient was transferred to the surgical intensive care unit (SICU). On day two, on examination, she was awake, aphasic, nodded yes/no to questions only, inconsistently followed commands, able to localize to noxious stimuli in right upper extremity and withdrew right lower extremity to noxious stimuli. Motor strength on right upper extremity was 1/5 and right lower extremity was 2.5. She was able to move left side spontaneously. Her clinical examination is consistent with her ischemic stroke in the left frontal parietal region. Repeat CT imaging revealed hemorrhagic transformation within the left basal ganglia and diffuse subarachnoid hemorrhage in the left cerebral sulci. Heparin therapy was subsequently discontinued on day two. Further diagnostic evaluation for stroke and hypercoagulable state yielded the following results: vitamin B12 108 pg/mL, homocysteine 34 µmol/L, methylmalonic acid 1.78 µmol/L, vitamin B6 8 ng/mL (Table 1).

**Table 1:** Laboratory assessment showed significant elevated levels of methylmalonic acid and homocysteine. Vitamin B12 and B6 were low. MCV: mean corpuscular volume, MCH: mean corpuscular hemoglobin, ALP: alkaline phosphatase, ALT: alanine aminotransferase, AST: aspartate aminotransferase

Test	Result	Units	Reference Range
WBC	10.8	K/CUMM	4.0 - 11.0
RBC	2.68	M/CUMM	4.7 - 6.1
Hemoglobin	11.7	gm/dL	13.5 - 17.5
Hematocrit	36	%	38.8 - 50.0
MCV	88.6	fL	80 - 100
MCH	35.1	pg	27 - 33
Platelets	345	K/CUMM	150 - 450
AST	10	U/L	10 - 40
ALT	12	U/L	7 - 56
ALP	86	U/L	44 - 147
Vitamin B6	2.8	µg/L	5.0 - 50
Methymalonic acid	1.78	µmol/L	0 - 0.40
Homocysteine	34	µmol/L	5.0 - 15
Lactate	3	mmol/L	<2
Vitamin B12	108	pg/ml	300 - 950

**Figure 4:**
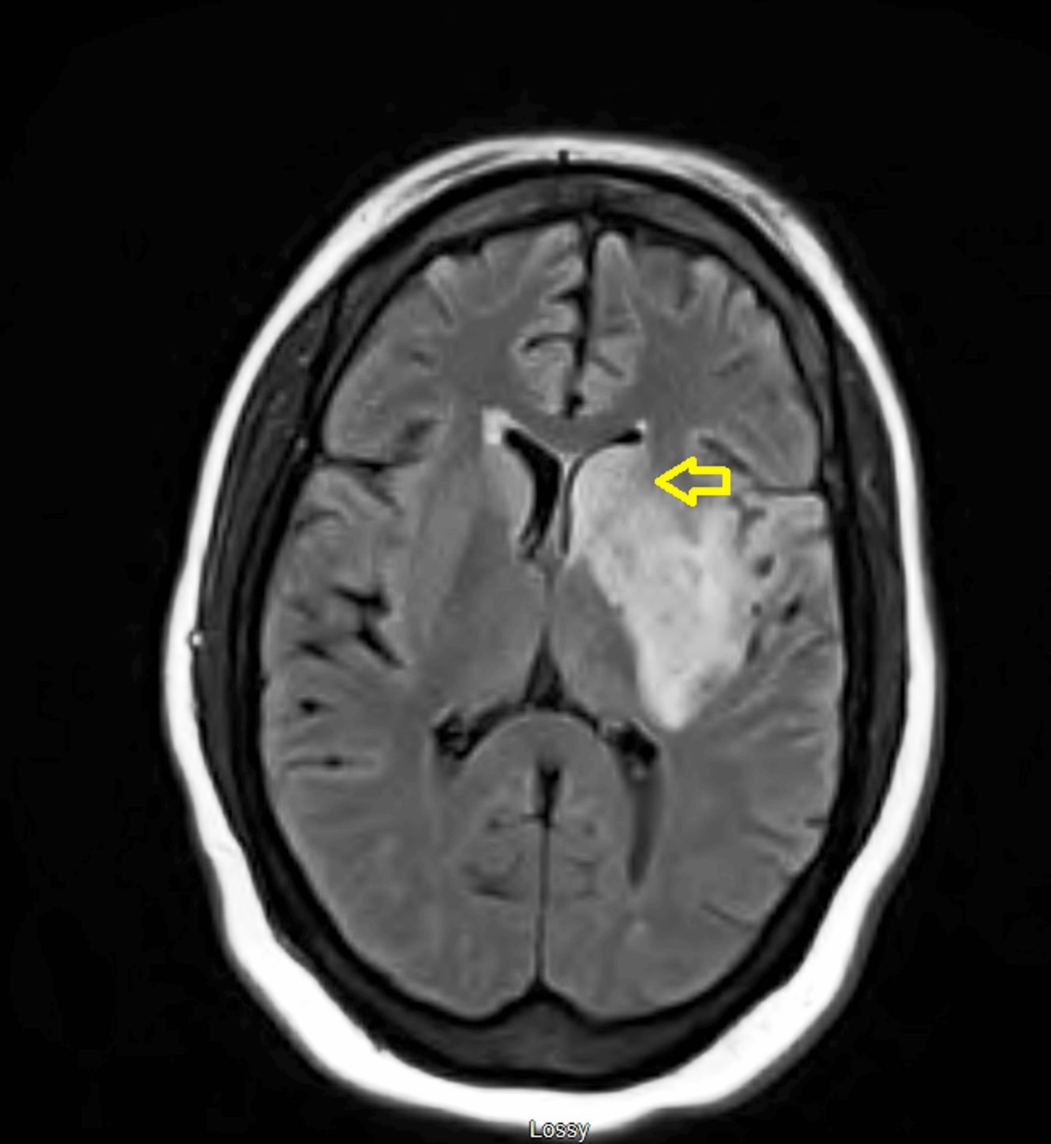
MRI showing infarction in the left insula and basal ganglia region with mass effect and midline shift to right.

Testing for factor V Leiden, prothrombin gene mutation, lupus anticoagulant, anticardiolipin antibodies, and beta-2 glycoprotein antibodies was conducted (results pending). She started on vitamin B12 1000 mcg intramuscular (IM) supplementation for seven days, vitamin B6 50 mg daily for two weeks and physical therapy/occupational therapy (PT/OT) was consulted. Heparin was reinitiated on day three when repeat CT head showed no progression of intracranial bleed. The risk of withholding IV heparin outweighed the benefit of using it, which led to the decision to restart anticoagulation with the agreement of Cardiology, Neurosurgery, and Neurocritical team. MRI showed evolving subacute infarction in the left insula and basal ganglia, with small areas of hemorrhagic transformation. Mass effect on the left lateral ventricle and 3 mm midline shift to the right, as shown in Figure 4.

She started showing improvement in her symptoms and became verbally responsive within a few days, although dysarthric. Her feeding tube was removed and a speech-language pathologist (SLP) evaluation recommended a barium swallow. Results showed severe oral dysphagia. Dysphagia level 1 pureed diet was recommended. Physical Medicine and Rehabilitation (PMR) was consulted and recommended inpatient rehab. She was discharged and transferred to inpatient rehab and remained admitted for almost four weeks. Later she was discharged to subacute rehab.

## Discussion

Nitrous oxide toxicity causes vitamin B12 deficiency, leading to neurological side effects related to central nervous system demyelination and subacute spinal cord degeneration. Nitrous oxide irreversibly inactivates vitamin B12 through oxidation. Consequently, the B12 metabolite cannot function as a coenzyme in converting methylmalonic acid and homocysteine. This results in elevated levels of both, which are better indicators of vitamin B12 deficiency [6]. Increased levels are associated with an elevated risk of venous thromboembolism, and hyperhomocysteinemia is associated with arterial thrombotic events [7]. Low levels of vitamin B12 are strongly associated with the risk of venous thromboembolism, with an OR of 10.76 independently, for idiopathic thrombosis [8]. In genome-wide association studies done in a community-based sample of the African American population in Mississippi, they reported 33 genetic variants located within two genomic regions (TCN1 and FUT2). A common loss-of-function variant in the TCN1 gene has been associated with lower vitamin B12 concentrations in African American populations. This variant, identified in a study by Hu et al. (2018), is prevalent among individuals of African descent and contributes to reduced levels of circulating vitamin B12. While the study did not find significant clinical consequences beyond lower B12 levels, the presence of this genetic variation, when combined with nutritional insufficiency or functional impairments in B12 metabolism, may increase the risk of severe vitamin B12 deficiency in certain populations [9]. 

In this case, she developed both arterial and venous thromboembolism. A hypercoagulable workup for common causes of arterial thromboembolism, including lupus and antiphospholipid antibody syndrome (the most common disease associated with both arterial [10] and venous thromboembolism), was negative. This was based on no history of first-trimester fetal demise, a normal coagulation profile upon admission, and negative antibody tests (anticardiolipin, lupus anticoagulant, beta-2 glycoprotein). Factor V Leiden mutation, Protein C, S and anti-thrombin 3 deficiencies were also negative.

The initial diagnosis of Guillain-Barré syndrome or chronic inflammatory demyelinating polyneuropathy (CIDP) may have been confounded by the neurological manifestations of vitamin B12 deficiency, which potentially contributed to her prolonged recovery phase. A plausible explanation for the pulmonary embolism could be an increased risk of thromboembolism within the pulmonary arteries themselves or the embolization of a thrombus originating from deep vein thrombosis (DVT) in the lower extremities. However, in this patient's case, DVT in the lower extremities was ruled out by duplex ultrasonography.

## Conclusions

Nitrous oxide should be taken into consideration while taking the social history in young adult populations because of its increased recreational use. Vitamin B12 deficiency, particularly when chronic, can lead to a hypercoagulable state, causing arterial and venous thromboembolism due to elevated methylmalonic acid and homocysteine levels. Nitrous oxide impairs vitamin B12 metabolism, which is why we may find near-normal vitamin B12 levels when evaluating these patients.

In patients presenting with neurological symptoms, a history of nitrous oxide abuse, and a thromboembolic event (including both arterial and venous thromboembolism), a high suspicion for vitamin B12 deficiency should be considered when evaluating for other possible differential diagnoses.

Treatment includes complete cessation of nitrous oxide use and initial intramuscular vitamin B12 for a few consecutive days, followed by a maintenance dose after laboratory confirmation. This will lead to symptomatic improvement in neurological symptoms and possibly decrease the incidence of other catastrophic events that can lead to lifelong disability. A limitation in our case was that we were unable to obtain an MRI spine to look for changes in the dorsal column associated with vitamin B12 deficiency.
